# The Scientific and Cultural Journey to Ovarian Rejuvenation: Background, Barriers, and Beyond the Biological Clock

**DOI:** 10.3390/medicines8060029

**Published:** 2021-06-08

**Authors:** E. Scott Sills

**Affiliations:** 1 Plasma Research Section, FertiGen CAG/Regenerative Biology Group, San Clemente, CA 92673, USA; ess@prp.md; 2 Department of Obstetrics & Gynecology, Palomar Medical Center, Escondido, CA 92029, USA

**Keywords:** fertility, platelets, cytokines, angiogenesis, menopause

## Abstract

Female age has been known to define reproductive outcome since antiquity; attempts to improve ovarian function may be considered against a sociocultural landscape that foreshadows current practice. Ancient writs heralded the unlikely event of an older woman conceiving as nothing less than miraculous. Always deeply personal and sometimes dynastically pivotal, the goal of achieving pregnancy often engaged elite healers or revered clerics for help. The sorrow of defeat became a potent motif of barrenness or miscarriage lamented in art, music, and literature. Less well known is that rejuvenation practices from the 1900s were not confined to gynecology, as older men also eagerly pursued methods to turn back their biological clock. This interest coalesced within the nascent field of endocrinology, then an emerging specialty. The modern era of molecular science is now offering proof-of-concept evidence to address the once intractable problem of low or absent ovarian reserve. Yet, ovarian rejuvenation by platelet-rich plasma (PRP) originates from a heritage shared with both hormone replacement therapy (HRT) and sex reassignment surgery. These therapeutic ancestors later developed into allied, but now distinct, clinical fields. Here, current iterations of intraovarian PRP are discussed with historical and cultural precursors centering on cell and tissue regenerative effects. Intraovarian PRP thus shows promise for women in menopause as an alternative to conventional HRT, and to those seeking pregnancy—either with advanced reproductive technologies or as unassisted conceptions.

## 1. Introduction

Cellular senescence is characterized by a degenerative process accompanied by poor tissue homeostasis and decline of organ function [1]. In clinical infertility practice, the ovaries generally are the first to suffer age impairment over time. Traditional teaching still embraces the basic tenet that females have a limited, non-replenishable oocyte endowment present at birth, which declines during adulthood and is eventually lost at menopause [2]. The basic concept of rejuvenation is not new, remaining topical yet contested. As currently offered, ovarian rejuvenation descends from a long line of medical efforts of varied efficacy that gained notoriety more than a century ago. In the public consciousness, any discussion of rejuvenation might have gained less notice had it not been for the developing study of gonadal physiology. Parallel improvements in surgical techniques also made human ovary tissue grafts workable from the late 1800′s, and testicular transplants for farm animals had been described even earlier [3]. Against this background, the mystique of ‘sex gland’ modulation was ideal for showcasing via the nascent clinical field of endocrinology.

It is now more accepted that human ovarian stem cells might persist into adulthood, retaining a capacity to differentiate into competent oocytes under appropriate conditions [4]. While no consensus exists regarding the exact signaling milieu necessary to elicit this replenishment, recent work suggests that pluripotency, self-renewal, and diminished follicular reserve in old age all may be subject to modulation [4,5]. This approach impacts reproductive medicine practice and patient counseling, as older ovaries not only supply fewer eggs but also yield cells of reduced competency—chiefly due to ploidy error. Moreover, the cumulative effect of age-sensitive mutations generally accelerates the exhaustion of ovarian reserve as reflected by diminished serum AMH and primordial follicle number [1,5,6]. When intraovarian PRP was administered to patients not seeking pregnancy [7], this non-pharmaceutical approach improved quality of life at least as effectively as standard hormone replacement therapy (HRT).

## 2. Cultural Contexts

Near the end of his life, novelist Thomas Mann (1875–1955) was among the first to portray gynecology with accuracy in popular literature. His short story, *The Black Swan*, describes a 50yr old widow yearning for recaptured youth. When menopause does reverse, her hopeful wish of rejuvenation seems granted. A heightened state of sexual arousal is perceived, where vitality and libido had previously withered. But her river of time did not really run backward. Instead, the high estrogen state was a symptom of advancing ovarian cancer. Death soon followed and the cruelty of nature’s hoax is revealed post-mortem [8].

William Butler Yeats (1865–1939) likewise had navigated his own creative drift and listlessness even earlier. At age 69, the Nobel laureate underwent an odd quasi-vasectomy seeking renewal. Praising that surgery, Yeats later acknowledged his recharged energy and sexual drive. That verve did not pass unnoticed in Dublin society, where he became known as the ‘gland old man’ [9]. Yeats’ literary output certainly charts an impressive post-operative flourish of late poems, which now rank among his most acclaimed works.

Not every man enjoyed such dramatic results. Sigmund Freud (1856–1939), who at age 67 had the same operation, obtained no impact on productivity. His quest for rejuvenation emerged concurrently with an awful struggle with oral cancer, entailing multiple painful surgical revisions. It was Freud’s belief that this vasectomy-variant might assuage his cancer, although he later admitted that ‘rejuvenation’ accomplished nothing [10]. Many less famous older men underwent this surgery in the early 20th century, in search of both mental and physical rejuvenation.

But what surgery was done? And why exactly was it thought so worthwhile? The concept that a type of vasectomy could yield such results traces back to the Vienna Academy and Prof. Eugen Steinach (1861–1944). What Steinach provided was a modification to standard sterilization, a familiar operation first reported in 1899 [9]. His hemi-vasectomy, now discredited, cut only one structure and conferred no contraceptive benefit. The belief was that this unilateral snip would shift the work of the affected testicle away from sperm production, and towards full-time production of male sex hormones. Steinach’s papers were widely accepted as key contributions to the discipline of the ‘science of fatigue’ [9]. Terminology eventually trapped the teacher; gentlemen did not merely have Dr. Steinach’s operation—they were ‘Steinached’ [10]. Tragically for women’s health of the day, ovarian X-ray treatment was considered the equivalent female procedure for what Prof. Steinach was doing for men in Vienna [10].

In 1930, Alice of Battenberg (1885–1969) a great-granddaughter of Queen Victoria, was suffering from chronic schizophrenia and arrived at Berlin’s Tegel Castle Sanatorium—the world’s first psychiatric clinic. A quiet childhood in Greece was upset by the turbulence of war and coup d’état, necessitating her family’s exile. There is no record that she had become distraught with menopause in her mid-40s, yet the clinic director sought the opinion of Freud who suggested ovarian X-ray therapy. This recommendation would have aligned with accepted techniques well known to Freud himself [11]. Of course, X-rays administered to the pelvis did nothing but increase medical problems for Princess Alice. The harms of ionizing radiation are now better understood in the post-nuclear age, especially cell death and endocrine collapse. Recent animal research commissioned to assess health risks for future female astronauts [12] has verified serious ovarian injury can occur even at trace radiation doses.

Another case is the California author Gertrude Atherton (1857–1948). She penned *Black Oxen* as a semi-autobiographical work, telling of an aging woman regaining youth with ‘glandular therapy’. While operative records are missing for contemporary critique, at age 66 Atherton did have some kind of experimental surgery and was convinced that rejuvenation had positive results for her [10].

After his death, Prof. Steinach slipped into obscurity despite having received multiple nominations for the Nobel Prize [13]. It became difficult to fit the topic of rejuvenation in the clinical canon, yet contemporary diarists recount him as a caring professional and dedicated researcher. Working ahead of then-accepted norms, his energies were focused on how best to integrate findings from highly innovative animal experiments into regular clinical practice [10]. Specifically, Steinach’s theories were among the first to posit how reproductive capacity and sexual phenotype could be changed if given proper ‘glandular’ inducements [9]. This offered crucial support to a new physiology of chemical/steroid regulation, eclipsing the old physiology centered on nervous system regulation [13].

Little persuasion was needed to show that if rats castrated before puberty could get back their vigor when given proper signals, then this same goal might also be possible for humans of a certain age. In the early 20th century, the door was thus open for uncritical belief in rejuvenation—and Steinach did not disappoint. He recorded how an innovative therapy using substances the healthy body was already making—not synthetic drugs or artificial chemicals—could recharge gonadal function [10]. By 1940, it was reasonable to expect modern science had at last unlocked the secret to rejuvenation. Along the way, Steinach had made professional connections with experts working in other disciplines, with rejuvenation research spinning off at least two clinical fields now well established: hormone replacement therapy (HRT) and transgender/sex reassignment surgery. Fortunately, an extensive literature exists to survey the progress in both domains.

Formal interest in HRT began in the 1960s, but reached its peak some thirty years later. Yet, thanks to Steinach, fundamental insights were charted even earlier in the 1930s. In 2002, clinical HRT trial data appeared on chronic postmenopausal symptoms from the Women’s Health Initiative (WHI). The WHI found synthetic HRT had many more negative effects than expected; HRT use dropped alarmingly after subsequent publicity altered prescribing patterns [14]. Yet HRT still dominates the pharmaceutical market with annual valuations consistently reaching billion-dollar levels [15,16,17,18].

Experience with rejuvenation also facilitated a paradigm shift where gender identity became reworked as a fluid target, orchestrated by sex steroids and their cyclic actions on brain and behavior [19]. Such transitions find many unsuccessful historical antecedents, some reaching back to ancient Rome. For example, Emperor Elagabalus (A.D. 204–222) is alleged to have offered a substantial reward to any physician who could give him a vagina [20], a story which secured a place for this early transgender figure—even if apocryphal. The film *The Danish Girl* (based on David Ebershoff’s 1930 novel of the same name) depicts the dean of sex reassignment therapy, Mangus Hirschfeld (himself a contemporary of Prof. Steinach) breaking barriers in hormone treatment. Although the protagonist, Lili Elbe (1882–1931), was a patient and noted artist who benefited from Hirschfeld’s skill in sex-change surgery, sadly her uterus transplant operation led to a fatal secondary sepsis. The partnership in the 1930′s between Steinach and Hirschfeld foreshadowed what would become transgender support [9], helping build a bridge between sex steroids and physical identifiers. Rejuvenation research found a role for composite ‘glandular action’ in shaping personal phenotype distinct from the accepted dogma that sex was simply a matter of presence of ovaries or testes [19,21]. As dots were connected among HRT, behavior, and appearance, early progress in rejuvenation produced gains in both quality of life and longevity [22]. As the published obituary for Steinach observed, ‘Not until the function of the ductless or endocrine glands was discovered, was there any hope that old age might be staved off’ [23].

## 3. New Directions

**Figure 1:**
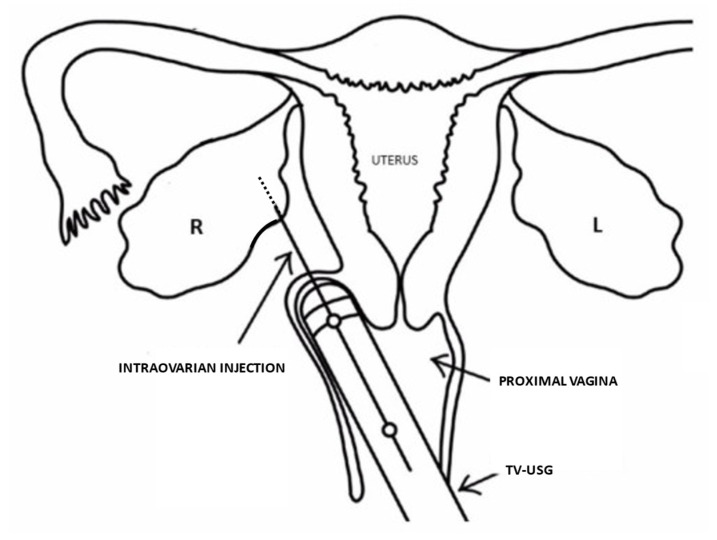
Injection of autologous PRP and/or condensed platelet-derived cytokines via transvaginal ultrasound guidance (TV-USG). Meaningful improvement after this ‘ovarian rejuvenation’ is contingent on in vitro platelet activation and release of soluble mediators to augment angiogenesis. These include epidermal growth factor, vascular endothelial growth factor, basic fibroblast growth factor, platelet-derived growth factor, transforming growth factor, platelet-derived angiogenesis factor, as well as several interleukins. After sample placement within ovarian tissue, serum anti-Mullerian hormone (AMH) level is measured over three months to assess potential changes in ovarian reserve. Post-treatment AMH patterns appear directly correlated with baseline platelet concentration [27]. Using standard IVF equipment, bilateral ovary PRP injection may be safely performed in ≤20 min without anesthesia or sedation [7].

The received wisdom concerning adult ovary function is being questioned, such that the precept of inevitable oocyte decline may have exceptions. Residual quiescent ovarian stem cells (OSCs) within aged or damaged ovaries now appear capable of differentiation towards competent oocytes in the context of proper signaling. This hypothesis came into sharper focus when animal research identified OSCs as a population of germline precursors not previously known. Working with adult murine ovarian tissue, such cells have been isolated and were successfully induced to yield developmentally competent oocytes [24]. Varied incubation and grafting techniques have been used to upregulate key meiosis-commitment genes necessary to permit oocyte formation [25]. But what are the ‘proper signals’ required to induce *de novo* egg development in adulthood? The answer seems to involve platelet-associated cargo proteins, which are numerous and still incompletely characterized [26]. The distinction between reported methods, where superiority or equivalence has not yet been sufficiently studied, rests on ovarian injection of activated platelets or their isolated, cell free supernatant. Such growth factors can improve tissue perfusion via intrastromal angiogenesis, and after intraovarian injection (see Figure 1) this could be expected to modulate oocyte competence by tempering intraovarian reactive oxygen species and/or mRNA upregulation coordinated by PRP-associated molecular signaling [27]. In addition to numerous cytokines released by activated platelets, the releasate also includes mitochondria capable of initiating multiple physiological responses [28].

A mitochondrial transfer process has also been reported in bone marrow-derived stromal cells and mesenchymal stem cells, providing beneficial metabolic effects for recipient cells [28,29,30]. Although this aspect of ovarian rejuvenation has (thus far) not been the topic of research, experimental findings described by others suggest that both cytokines and mitochondria available after platelet activation are appropriate targets for closer study.

As repair and regenerative processes organized by platelet cytokines in somatic cells are poorly understood, their precise effects in adult human ovarian (germinal) tissue are even less known. Yet if stem cells are indeed present in the postnatal ovary, might PRP placed there evoke undesirable tumorigenic changes? While this might be a theoretical risk, thus far it has not been observed in any clinical context. Because platelet cytokines are ligands that attach to cell membrane receptors—not the nucleus—their mechanism of action is unlike trophic hormones [31]. Further reassurance comes from work in other human tissue treated with PRP, where accelerated growth of healthy cells was noted without triggering malignant transformation [32]. This probably explains why multiple intraovarian PRP methods have been used with no adverse events resulting from any technique.

Perhaps less obvious is the worry that effective ‘ovarian rejuvenation’, if utilized with wide application might enhance fertility so substantially as to alter the world population. This impact is unlikely, given the present condition where those countries with the highest IVF uptake nevertheless continue to register among the lowest national birthrates. Crucially, the ever-growing number of patients seeking relief from menopause will always far exceed the infertility case sub-group, even though both populations stand to benefit from intraovarian PRP.

Experience with autologous PRP is clearing a new non-synthetic pathway to reset the ovarian equipoise prevalent before perimenopause. Improvement in follicular development, hormone balance, and successful pregnancy after intraovarian PRP have all been achieved [5,7,33,34], but the question of duration of effect is unanswered. This represents a key limitation, but as with traditional HRT dosing [14], conditions resolved by intraovarian PRP will likely also require maintenance therapy. Unfortunately, no longitudinal data exist regarding sustained ovarian capacity improvement following PRP treatment, although this research is planned.

## 4. Conclusion

While the link between platelet-derived cytokines and/or mitochondria and reproductive potential awaits validation by RCTs, several case papers already offer views to what may be clinically attainable. For example, experts in Greece [33] described a successful alternative intraovarian PRP treatment leading to the first report of pregnancy in menopause (age 40yrs). Although the patient did not continue to delivery, the report nevertheless advances the rejuvenation field as a proof-of-concept work. In addition, embryo genetic imbalances may benefit from upstream correction after ovarian treatment with condensed platelet cytokines, resulting in healthy term livebirth [34]. These are promising results and should serve as an early foundation to encourage additional research.
